# Bacteriomes in lesions of pulmonary tuberculosis and its association with status of *Mycobacterium tuberculosis* excretion

**DOI:** 10.1186/s12866-022-02698-5

**Published:** 2022-11-23

**Authors:** Weili Du, Yingli Zhao, Li Zhang, Jialu Che, Zichen Liu, Kun Li, Nanying Che

**Affiliations:** Department of Pathology, Key Laboratory for Drug Resistant Tuberculosis Research, Beijing Chest Hospital, Capital Medical University, Beijing Tuberculosis and Thoracic Tumor Research Institute, Beiguandajie 9#, Tongzhou Dist, BeijingBeijing, 101149 China

**Keywords:** Pulmonary TB lesions, Status of MTB excretion, Lung bacteriomes, Bacterial MetaCyc functions

## Abstract

**Background:**

Bacteria in lung play an important role in sustaining lung health. Understanding the characteristics of bacteriomes in lesions of pulmonary tuberculosis (TB) patients, who excrete *Mycobacterium tuberculosis* (MTB), is important for TB prevention and effective treatment.

**Methods:**

In this study, bacteriomes in lesions from TB patients excreting bacteria (TB-E) and those from TB patients not excreting bacteria (TB-NE) with matched normal lung tissues (NT) were compared by 16S rRNA sequencing. Bacterial MetaCyc functions in TB lesions were also predicted by PICRUSt2 tool.

**Results:**

Alpha diversity of bacteria, including Chao 1 and Shannon indexes, for TB-E was significantly higher than those in TB-NE and NT; while for TB-NE group, Chao 1 index was higher than that in NT group. Predominant phyla in TB lesions and NT were *Proteobacteria*, *Actinobacteria*, *Firmicutes*, and *Bacteroidetes*, but analysis of similarity (ANOSIM, *p* < 0.001) revealed significantly different bacterial compositions among TB-E, TB-NE and NT samples. As for bacteriomes in TB lesions, a strong association (ANOSIM, *p* < 0.001) was observed with the status of MTB excretion. Indicator genera identified in TB-E and TB-NE demonstrated distinctive micro-ecological environments of TB lesions from patients with different clinical manifestations. Co-occurrence analysis revealed a densely-linked bacterial community in TB-NE compared to that in TB-E. MetaCyc functions responsible for menaquinone synthesis and chorismate metabolism that could potentially impact the persistent-state and nutrient metabolism of MTB were enriched in TB-E samples. While in TB-NE samples, enrichment of bacterial MetaCyc function responsible for heme b synthesis might contribute to TB pathology through ferroptosis.

**Conclusion:**

Bacteriomes and their MetaCyc functions in TB lesions are elucidated, and they are associated with status of MTB excretion among pulmonary TB patients. These results serve as a basis for designing novel strategies for preventing and treating pulmonary TB disease.

**Supplementary Information:**

The online version contains supplementary material available at 10.1186/s12866-022-02698-5.

## Background

Pulmonary tuberculosis (TB), caused by *Mycobacterium tuberculosis* (MTB) infection, is transmissible through the airborne route [1]. Only patients with sputum culture-positive are considered infectious, and they can threaten public health. Although antibiotic treatment is the fundamental therapeutic regimen for pulmonary TB according to the World Health Organization (WHO) [2], the conversion time of sputum culture varies greatly between individuals [3].

Organized granuloma is a typical lesion of pulmonary TB patients. TB lesions are reservoirs for MTB survival in the lung. Physiological and biochemical conditions of TB lesions, such as penetration of antibiotic drugs, oxygen tension, acidic pH, and lipid abundance, would impact MTB’s status of persistence, replication and transmission [4]. When the center of the granuloma liquefies and ruptures, MTB bacilli become extracellular, and it can be released into the air through coughing, talking, or sneezing, which directly results in sputum culture-positive outcomes. Negative conversion of sputum culture is a crucial indicator to monitor patient’s prognosis during antituberculosis treatment. Many non-antibiotic factors, including history of smoking [5], chest cavity [3], body mass index (BMI) [6], etc., have been reported to be significantly associated with the time of sputum culture conversion. However, it is the status of MTB excretion in TB lesions that ultimately determines the conversion of sputum culture results.

Pulmonary inherent bacteria have been proven essential both in healthy and diseased status of the lung [7, 8]. Furthermore, it can promote the host’s resistance to MTB colonization through regulating mucosal-associated invariant T (MAIT) cells in the lung of mice model [9]. Besides the physiological and biochemical impacts of TB lesions on MTB colonization [4], lung bacteria may be another important factor that can affect the growth behavior of MTB. Many studies have investigated the lung bacteria of pulmonary TB patients, but they were carried out using sputum [10–12] or bronchoalveolar lavage fluid (BLF) samples [13, 14]. However, microbiota in sputum and BLF samples cannot exactly represent the entire inhabitants in lung, specifically in TB lesions. So far, the characteristics of bacteriomes in TB lesions have not been clearly investigated yet. Taken together, whether the inherent bacteria in TB lesions contribute to the conversion of MTB excretion status is poorly understood.

In the current study, TB lesions and normal lung samples were respectively collected from pulmonary TB and lung cancer patients, who have undergone surgical treatment. Sputum culture results of MTB for all enrolled pulmonary TB patients were also collected. We explored the characteristics of bacterial composition in TB lesions and normal tissues using 16S rRNA sequencing method to 1) compare the distinctive features of bacterial profiles in TB lesions and normal lung tissues, and 2) analyze the association between pulmonary bacteria and MTB excretion status in TB patients. Metabolic functions of bacteria in TB lesions were also predicted using the phylogenetic investigation of communities by reconstruction of unobserved states 2 (PICRUSt2) tool to reveal the correlations between predicted functions and status of TB lesions.

## Methods

### Clinical characterization of the participants

Formalin fixed and paraffin embedded (FFPE) TB lesions and normal lung tissues (NT) from pulmonary TB patients and lung cancer patients, who have undergone surgical treatment, were obtained in the Department of Pathology, Beijing Chest Hospital. The clinical indications for biopsy in patients with tuberculosis were that the anti-tuberculosis drug treatment was ineffective and the lesions were relatively localized. The biopsy samples used in this study were obtained from lung through surgical operation for pathological morphological and molecular diagnosis of TB disease. The standard for the diagnosis of pulmonary TB disease was to meet any of the followings: 1) mycobacterial culture for sputum was positive; or 2) TB-PCR of *IS6110* fragment for TB lesion was positive. Patients whose sputum culture results were positive were classified as TB patients excreting bacteria (TB-E) or TB patients not excreting bacteria (TB-NE) if their sputum culture results were negative but TB-PCR for TB lesions were positive. Totally, 82 pulmonary TB patients were obtained, including 40 TB-E and 42 TB-NE cases, respectively. Additionally, 18 lung cancer patients, whose NT samples > 2 cm distal to tumor lesions, were enrolled as bacterial baseline. All participants had not taken any medications for human immunodeficiency disease before samples collection.

### DNA extraction and processing

According to the hematoxylin–eosin (HE) staining results, the normal lung tissues surrounding the TB lesions were removed from the FFPE samples, and 0.5–1.0 cm^2^ lesion areas were retained. These HE-stained lesion areas showed necrotic or non-necrotic granulomatous inflammation with visible epithelial cells, chronic inflammatory cells, and/or Langhans giant cells surrounded by fibrous connective tissue. NT samples with 0.5–1.0 cm^2^ areas were selected for this study, and HE-stained NT samples showed normal lung lobule structure. Twenty Sects. (4 μm) of TB lesions or NT samples were used to extract DNA using QIAamp DNA FFPE Tissue Kit 56,404 (QIAGEN, Hilden, Germany), according to the manufacturer’s instructions. The concentration of the DNA was measured using a NanoDrop 2000 spectrometer (Thermo Fisher Scientific, Waltham, United States). The integrity of DNA was evaluated by 1% agarose gel electrophoresis.

Amplification of 16S rRNA V4 hypervariable region was carried out with primer sets, 515F (5’-GTGCCAGCMGCCGCGGTAA-3’) and 806R (5’-GGACTACHVGGGTWTCTAAT-3’), with barcodes at the 5’ end of the forward primers. The PCR reaction system was 50 μL, with 1.5 μL primer sets (10 μmol/L), 25 μL 2 × KAPA HiFi HotStart ReadyMix (Kapa Biosystems, MA, United States) and 23 μL extracted FFPE DNA. The PCR protocol included: initial denaturation at 95 ℃ for 3 min, 30 cycles of denaturation (20 s at 98 ℃), annealing (15 s at 55 ℃) and extension (15 s at 72 ℃), and a final extension at 72 ℃ for 10 min. The PCR reaction was triplicated for each sample, and the three PCR products were pooled together and purified through 2% agarose gel electrophoresis. The concentration of purified PCR products was quantified using a Qubit 4.0 fluorometer (Life ABI, Waltham, United States) before DNA library construction. Agilent 2100 bioanalyzer (Agilent Technologies, Palo Alto, California) was used to confirm fragment size and distribution of the constructed DNA library. The library was barcode indexed with the NEB Next Ultra DNA Library Prep Kit for Illumina (New England Biolabs, United States), and sequenced on Illumina MiSeq PE 250 machine.

### 16S rRNA gene analysis

After sequence demultiplex, the raw data were analyzed using QIIME v1.9 [15]. The sequences were quality trimmed and filtered (fastq_maxee = 0.5) using QIIME default parameters, and qualified sequences were clustered into OTUs (operational taxonomic unit) by the closed-reference OTU picking methods at 97% sequence similarity using USEARCH v7.0.1090 [16]. The filtered OTUs with the number of sequences < 0.005% of the total were discarded [17]. Greengenes database (August 2013 release) was referred to annotate bacterial taxa. OTUs that mapped to the mitochondria and chloroplasts were discarded. All samples were then rarefied to the same sequence number, according to the number in the sample that yielded the fewest sequences. The final rarefied OTU table, providing the abundances of bacterial taxa in each sample, was used for downstream analyses. QIIME was also used to create α-diversity indexes, including Chao1 richness, Shannon, and Good’s coverage, as well as β-diversity index (Bray–Curtis distance metrics). PICRUSt2 was used to generate a MetaCyc metabolic pathway to predict the functions of 16S rRNA sequences in each sample [18].

### Statistical analysis

Bacterial variety among different pulmonary tissue categories was visualized by principal coordinates analysis (PCoA), based on Bray–Curtis distance using the “ggplot2” package for R [19]. To statistically verify the bacterial clustering in the PCoA analysis, the analysis of similarity (ANOSIM) algorithm was utilized. Heatmap with K-means clustering analysis was made using “pheatmap” package for R [20]. The Venn diagram was drawn using the “VennDiagram” package [21]. Bacterial indicators in different categories were predicted using “indicspecies” packages, with *n* = 999 random permutations. Analysis of variance (ANOVO) and Tukey’s test together were used to calculate statistical differences of α-diversity indexes between different tissue categories. Two-sample proportion z test was used to calculate the statistical differences of bacterial species and MetaCyc functions between two categories. These analyses above were performed in the R software program (v.3.2.1; http://www.r-project.org).

## Results

### Clinical characteristics of pulmonary TB patients

Detailed clinical and demographical characteristics of pulmonary TB patients are listed in Table 1. Eighty two pulmonary TB lesions from 82 patients were obtained, including 40 TB-E and 42 TB-NE cases, respectively. The number of patients who had cough, fever, night sweats, weight loss, or cavitation on chest CT in the TB-E group showed no statistical differences compared to those in the TB-NE group. Patients in the TB-NE group were 9.4 years younger than those in the TB-E group (*p* = 0.005). The proportion of patients with hemoptysis symptoms in the TB-E group was significantly lower than that in the TB-NE group (*p* = 0.003). A major proportions of TB patients in TB-E (72.5%, 29/40) and TB-NE (65.9%, 27/42) groups have received standard HREZ regimens (ie isoniazid, rifampicin, pyrazinamide and ethambutol). Also, the numbers of patients receiving standard HRZE regime showed no difference (*p* = 0.684) between the two groups. It should be noted that *IS6110* amplifications of the 82 TB lesion tissues were all positive, though acid fast bacilli (AFB) positive ratios of TB lesion tissues from TB-E and TB-NE patients were 67.5% (27/40) and 71.4% (30/42). Also, we collected 18 NT samples from 18 lung cancer patients to be used as the bacterial baseline.

**Table 1:** Characteristics of participants with pulmonary tuberculosis

Variable	TB Culture Results, No. (%)		*p* Value^a^
	Positive (*n* = 40)	Negative (*n* = 42)	
Age, average (std)	49.1 ± 16.8	39.7 ± 12.5	0.005
Male	27 (67.5)	19 (45.2)	0.071
Any cancer	3 (7.5)	0 (0)	0.223
Cough	14 (35.0)	21 (50.0)	0.250
Hemoptysis	1 (2.5)	12 (28.6)	0.003
Fever	7 (17.5)	9 (21.4)	0.865
Night sweats	3 (7.5)	7 (16.7)	0.352
Weight loss	9 (22.5)	5 (11.9)	0.327
Tissue AFB positive	27 (67.5)	30 (71.4)	0.884
Cavitation on chest CT	7 (17.5)	14 (33.3)	0.165
Therapeutic regimen (HRZE^b^)	29 (72.5)	27 (65.9)	0.684

^a^Calculated based on the Pearson χ^2^ test with correction for Yates’ continuity or the Fisher exact test (for expected count < 5)

^b^isoniazid-rifampicin-pyrazinamide-ethambutol

### Distinct bacterial compositions among TB-E, TB-NE and NT samples

**Fig. 1:**
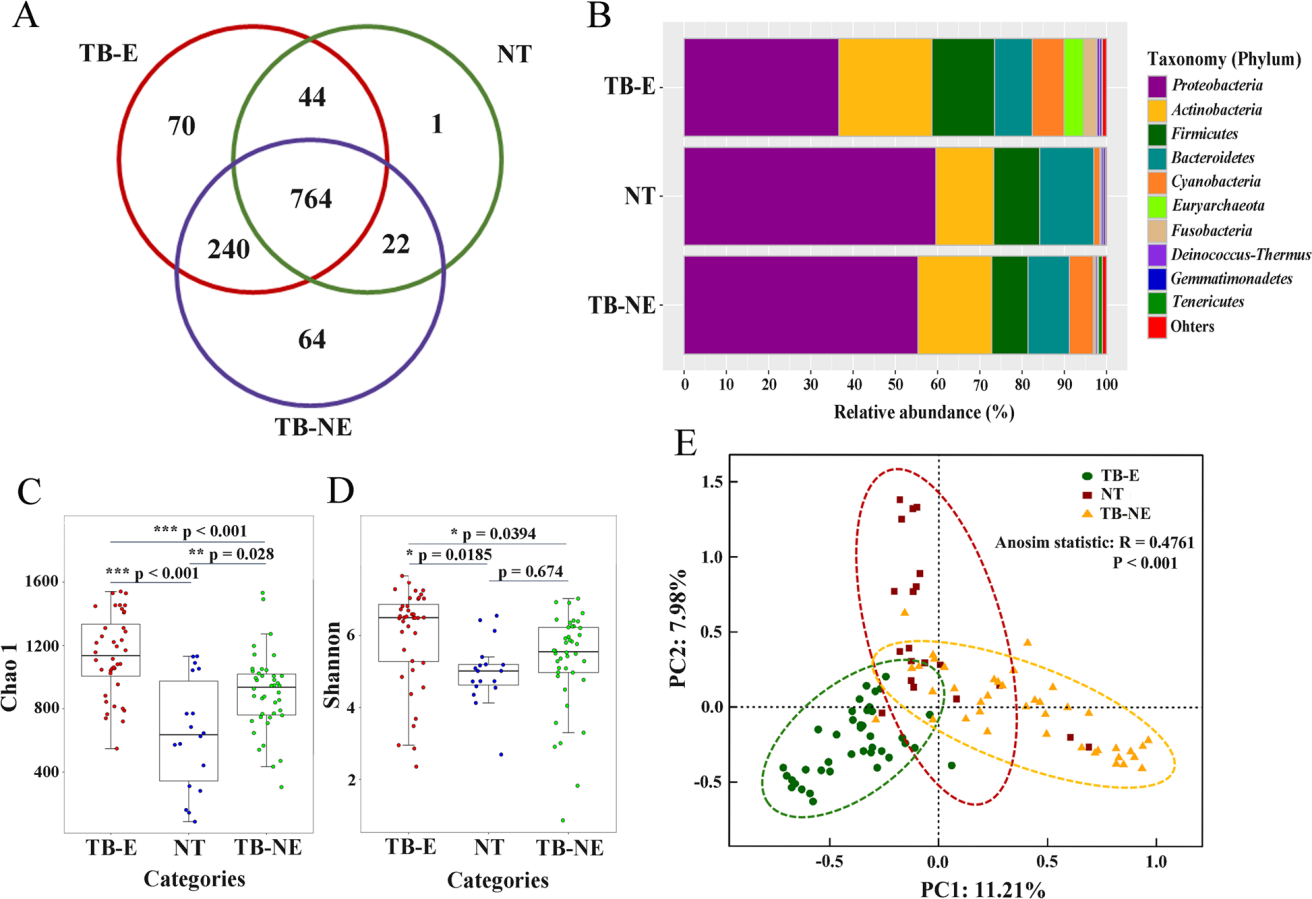
TB-E, TB-NE and normal lung tissues (NT) have distinct bacterial diversities. **A** Venn diagram of the exclusive and shared OTUs. **B** Average relative abundances of phylum-level taxa showing the distribution of bacterial compositions. **C** and **D** Box plot of Chao1 and Shannon index of the three tissue categories. **E** Principal coordinate analysis (PCoA) based on Bray–Curtis dissimilarity displaying the bacterial variations. Statistical differences are calculated using the analysis of similarity (ANOSIM) algorithm. The dotted circles indicate the 95% confidence intervals. TB-E: Lesions from TB patients excreting bacteria; TB-NE: Lesions from TB patients not excreting bacteria

A data set of 16S rRNA gene fragments with 5,670,646 valid sequences was obtained after quality control and filtration of DNA fragments from mitochondria and chloroplast. Sequence numbers for each sample ranged from 33,658 to 65,701. To avoid the bacterial discrepancy caused by different sequencing depths, all sample sequences were rarefied to 33,658 before further analysis. Based on the 97% similarity threshold, 1,205 OTUs were clustered from these sequences. Venn diagram showed that 63.40% (764/1,205) of OTUs were shared by all the samples (Fig. 1A). The proportions of specific OTUs in the TB-E group (5.81%, 70/1,205) and the TB-NE group (5.31%, 64/1,205) were similar, but both much higher than that in the NT group (0.08%, 1/1,205) (Fig. 1A). The single specific OTU in NT group was an uncultured bacterium belonged to genus *Rubellimicrobium*. *Proteobacteria*, *Actinobacteria*, *Firmicutes*, *Bacteroidetes*, and *Cyanobacteria* were the major phyla observed in human lung tissues, which accounted for 94.4% of the sequences in total (Fig. 1B). Additionally, the archaeal phylum *Euryarchaeota* was also detected, and it was more predominantly in TB-E samples (100%, 40/40) than that in TB-NE samples (45.2%, 19/42). Phylum *Euryarchaeota* was rarely reported in previous TB microbiome studies using sputum or BAL samples. Within the TB-NE group, *Proteobacteria* accounted for a higher sequence number (*p* < 0.001), while *Firmicutes* accounted for a lower sequence number (*p* = 0.006) when compared to the TB-E samples. A larger proportion of phylum *Cyanobacteria* were observed in TB-E (*p* = 0.003) and TB-NE (*p* = 0.014) groups than that in NT samples. In genus level, predominant bacteria in TB lesions and NT samples were *Pseudomonas*, *Bacteroides*, *Mycobacterium*, *Sphingomonas*, *Ottowia* and *Cutibacterium*. However, NT group was enriched with *Pseudomonas* and *Bacteroides*; while TB-E and TB-NE were both enriched with *Mycobacterium*.

Alpha diversity of pulmonary bacteriomes was evaluated by Chao 1 and Shannon indices (Fig. 1C-D). Chao 1 and Shannon indices of TB-E were both significantly higher than those of TB-NE and NT groups (ANOVA, *p* < 0.05). When comparing the alpha diversity of the TB-NE group with that of the NT samples, Chao 1 index was statistically higher (*p* = 0.028), but Shannon index showed no significant difference (*p* = 0.674). Principal coordinates analysis (PCoA) demonstrated that bacterial compositions distinctly clustered in line with the three different tissue categories (Fig. 1E). This separation was further confirmed by analysis of similarity (ANOSIM) (*p* < 0.001). These results indicate that bacterial composition in TB lesions is significantly different from that in normal lung tissue, and is strongly associated with the MTB excretion status.

Totally, 49 OTUs assigned to genera *Mycobacterium* were defined from bacterial 16S rRNA sequences. *Mycobacterium* accounted for a proportion of 0–13.94% of the total 16S rRNA sequences in TB lesions, but their relative abundance showed no significant difference (*p* = 0.186) between TB-E and TB-NE groups.

**Fig. 2:**
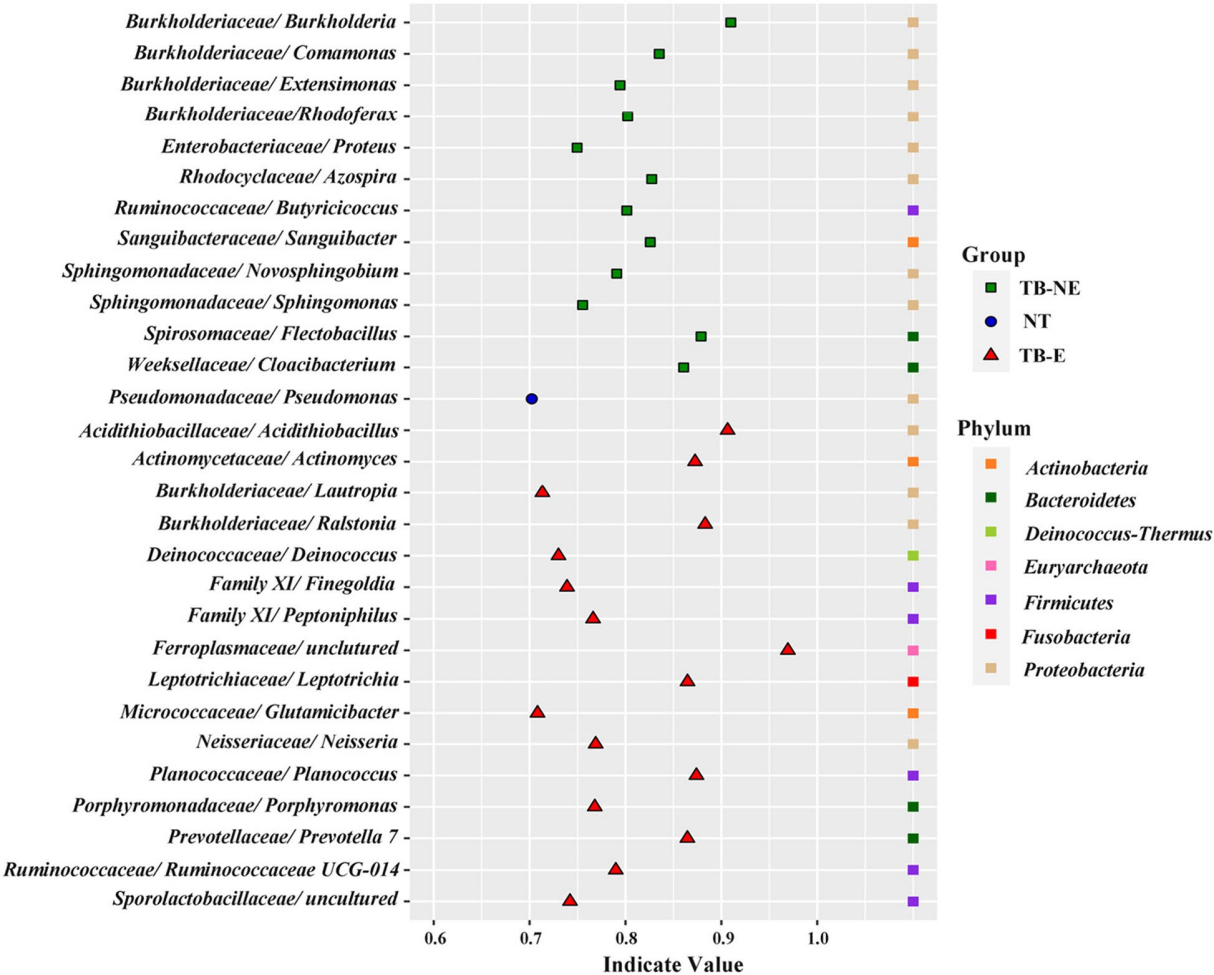
IndVal analysis showing indicator genera in TB-E and TB-NE samples. Genera taxa with an indicator value > 0.70 and *p* < 0.05 are identified as indicators

Indicator genera can reflect the distinctive microenvironments in various habitats. Indicator genera in TB lesions and NT samples were identified by IndVal analysis (Fig. 2). With an indicator value > 0.70 and *p* < 0.05, there were respectively 16, 12 and 1 indicator genera in TB-E, TB-NE and NT samples. Indicator genera in TB lesions belonged to *Actinobacteria*, *Bacteroidetes*, *Deinococcus*-*Thermus*, *Euryarchaeota*, *Firmicutes*, *Fusobacteria* and *Proteobacteria*. Significantly, 66.7% (8/12) indicator genera in TB-NE group were *Proteobacteria*. An uncultured archaea affiliated to *Ferroplasmaceae* was also observed in TB-E group as an indicator genus. The single indicator genus in NT samples was *Pseudomonas*, which was not the specific taxa *Rubellimicrobium* described above.

### Co-occurrence analysis of bacterial interactions in TB lesions

**Fig. 3:**
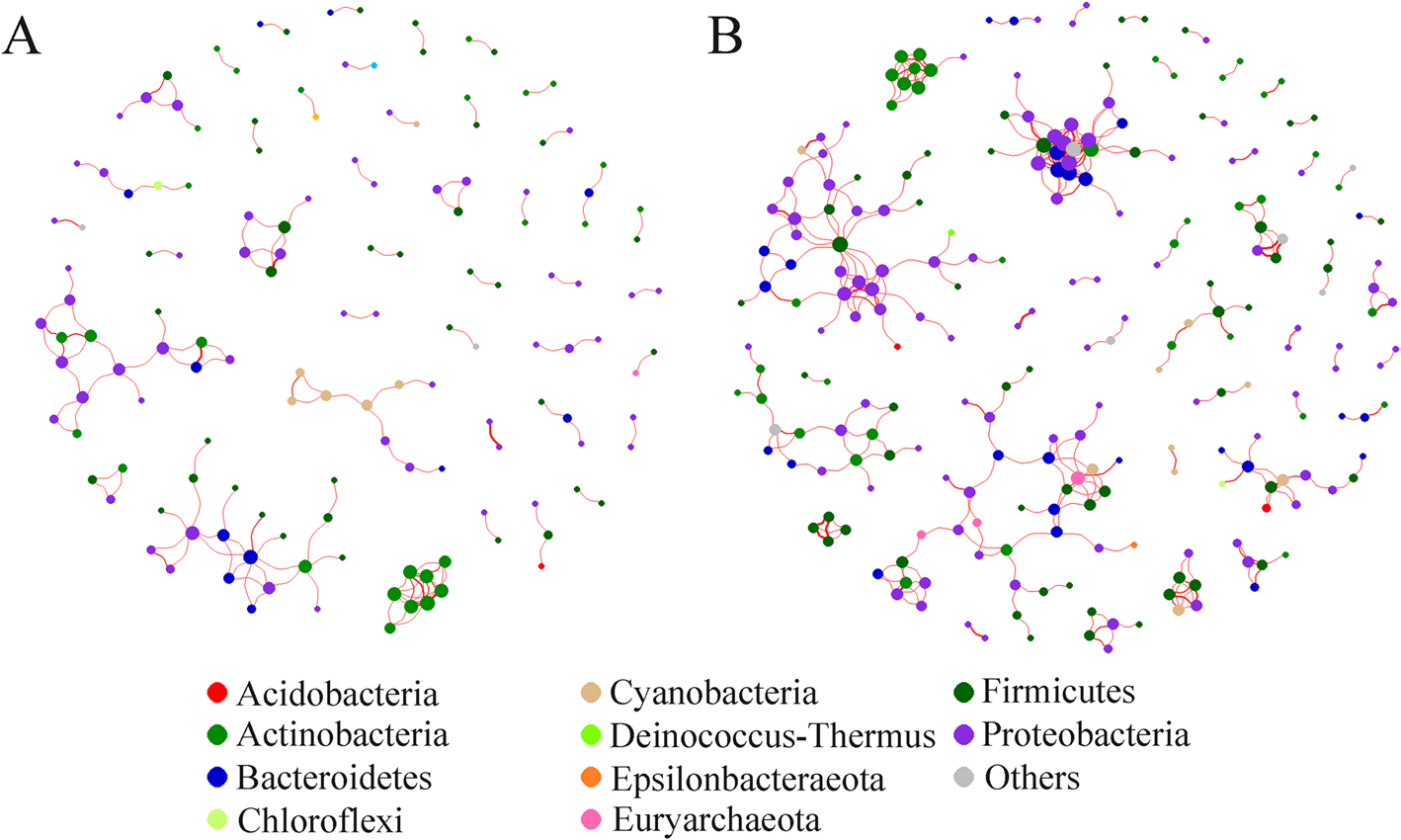
Network of co-occurring genera showing the bacterial interactions in TB-E (**A**) and TB-NE (**B**). Each connection indicates a strong (Spearman’s ρ > 0.6) and significant (*p* < 0.01) correlation. The size of each node is proportional to the number of connections, and the edge thickness is proportional to the weight of each correlation

The specific bacterial co-occurring networks, including strong (Spearman’s ρ > 0.6) and significant (*p* < 0.01) correlations, were generated in the TB-E and the TB-NE samples to explore how bacteria were potentially connected with each other (Fig. 3). Nodes in the network were assigned to 10 bacterial phyla, among which two phyla (*Proteobacteria* and *Actinobacteria*) were widely distributed, together accounting for 56.12% of all nodes. However, no taxa showed statistical correlation with the genera *Mycobacterium* in both TB-E and TB-NE groups. Topological parameters were further calculated to describe the complex pattern of bacterial inter-relationships in TB-E and TB-NE groups. The number of nodes, edges, clustering coefficient, average path length, network diameter and degree in TB-E group were respectively 142, 136, 0.505, 2.401, 6 and 1.915, which were all lower than those in the TB-NE group (Table 2). The edge and node numbers in TB-E group were less than that in TB-NE group, for which the average path length was also lower, suggesting that bacteria in MTB non excreting lesions were less connected but those correlated with each other were quite closely connected. The smaller average path length of network for TB-E group indicated a “small-world” property, which was believed to have a more effective and close bacterial connections to response quickly to external perturbations [22].

**Table 2:** Topological properties of co-occurring networks for the TB-E and TB-NE groups

Group	Nodes	Edges	Modularity (MD)	Clustering coefficient (CC)	Average path length (APL)	Network diameter (ND)	Average degree (AD)
TB-E	142	136	0.907	0.505	2.401	6	1.915
TB-NE	234	346	0.871	0.618	3.361	9	2.957

### Bacterial MetaCyc functions identified in TB lesions

**Fig. 4:**
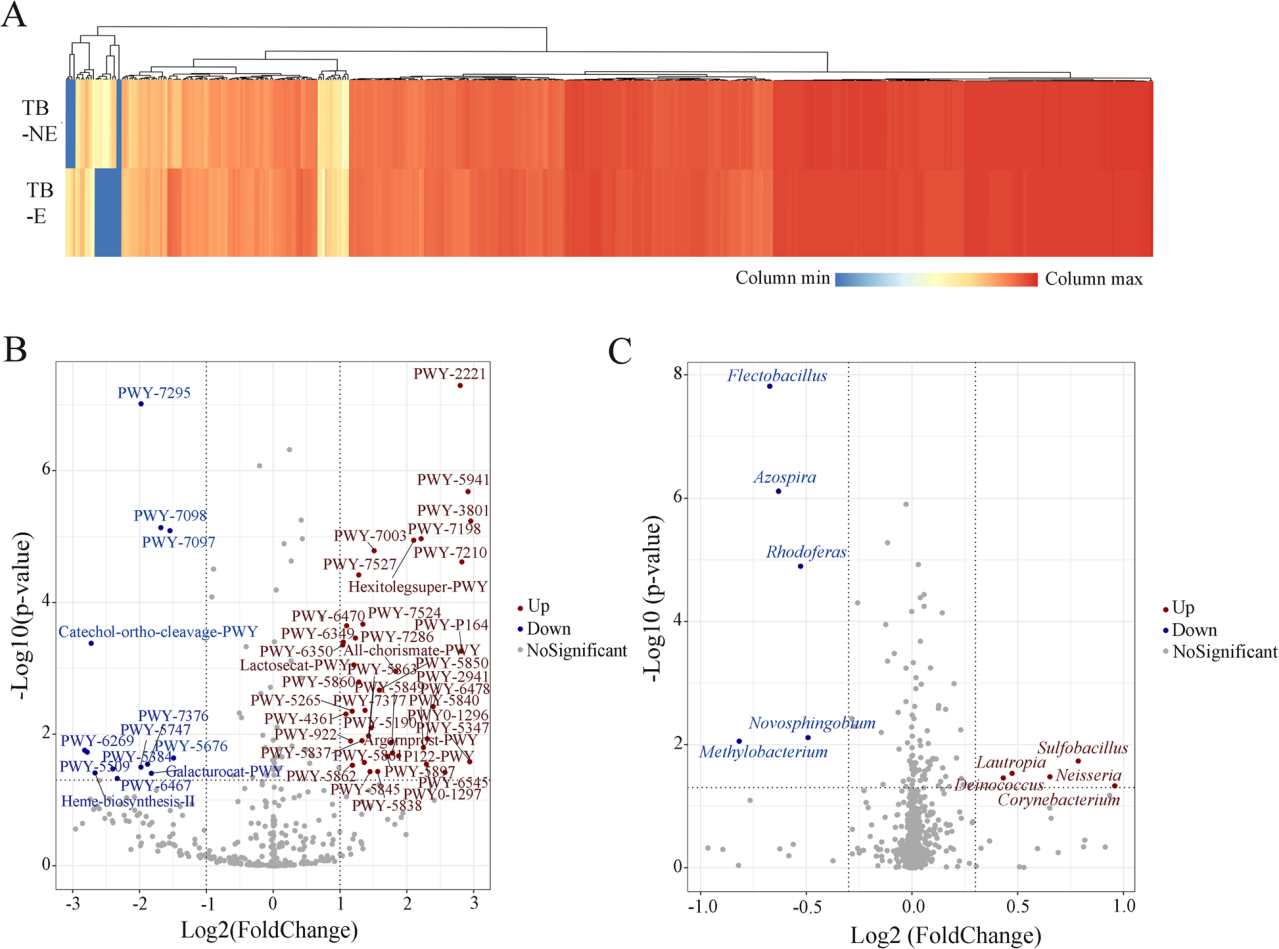
Bacterial metabolic functions predicted by PICRUSt in TB lesions. **A** Heatmap demonstrating K-means clustering of 449 metabolic pathways across TB-E and TB-NE samples. **B** Volcano plots showing bacterial metabolic pathways that differ in TB-E compared with TB-NE. **C** Volcano plot demonstrating the genera with striking differences between TB-E and TB-NE sample types. A two-sample t test was used to calculate *p* values in (**B**) and (**C**)

Totally, 449 bacterial MetaCyc functions of pulmonary bacteria were predicted using the PICRUSt2 tool based on 16S rRNA sequencing data from TB lesions (Fig. 4A, Supplemental Table 1), mainly including energy metabolism, environmental and information processing, genetic information processing, and cellular processing. These bacterial MetaCyc functions were responsible for maintaining the basic physiological activities and they were widely distributed in taxonomically distinct bacteria. Therefore, we mainly focused on 55 bacterial MetaCyc pathways that were statistically different between TB-E and TB-NE samples (Fig. 4B, Supplemental Table 2).

Forty-one bacterial MetaCyc pathways with statistically richer levels in TB-E samples included menaquinone biosynthesis, chorismate metabolism, penicillin resistance, hexitol degradation, nucleic acid processing etc. In TB-NE samples, 14 bacterial MetaCyc pathways that were responsible for vanillin and vanillate degradation, catechol degradation, D-galacturonate degradation, heme b biosynthesis, adenosylcobamide biosynthesis etc., were significantly enriched. Ten genera were markedly different between the two groups (Fig. 4C), but they could not explain the discrepancy of bacterial MetaCyc functions described in Fig. 4B and Table S2. This is reasonable because bacterial composition is generally decoupled from bacterial metabolic functions, and each single metabolic function is usually performed by multiple coexisting, taxonomically distinct bacteria [23]. Additionally, most bacterial taxa in TB lesions were uncultured or unidentified, and detailed information about their metabolic functions is still unknown.

## Discussion

Lung bacteriomes play an important role in TB pathophysiological processes and clinical manifestations, but very few studies have investigated the bacteriomes in lower respiratory tract using lung tissues. Different from previous investigations on pulmonary bacteria in TB patients by using sputum or BAL samples [11–14, 24], we collected lung tissues to reflect the characteristics of lung bacteriomes. This study initially explored the characteristics of bacteriomes in TB lesions and normal lung tissues, and evaluated its association with MTB excretion status.

Microenvironmental conditions in the human lung, such as nutrient availability, oxygen tension, activation of inflammatory cells, and host epithelial cell interactions, will change greatly during the formation of granuloma lesions [7]. TB lesions provide new ecological niches for bacteria, resulting in a new balance of bacterial immigration, elimination, and bacterial relative production rates [25]. TB lesions are places where MTB grows and host anti-TB immune responses occur. Bacteria in TB lesions can directly interact with MTB and participate in anti-TB immune responses [9]. Dominant phyla, with relative abundance from high to low, observed in the TB lesions were successively *Proteobacteria*, *Actinobacteria*, *Firmicutes*, *Bacteroidetes*, and *Cyanobacteria*. Ticlla and her colleagues have reported *Firmicutes*, *Proteobacteria*, *Bacteroidetes*, *Actinobacteria* and *Fusobacteria* as the major phyla order in sputum samples [12]. This difference is reasonable because microbes from the upper respiratory tract and the oral cavity can contaminate sputum samples inevitably during the expectoration process, which will bias the microbial profile in sputum. In BAL samples, the relative abundance of bacteria from high to low is *Actinobacteria*, *Firmicutes*, *Proteobacteria*, *Bacteroidetes* and *Fusobacteria* [13], which is different from our study. Bacteria load in BAL is much lower than that in lung tissues [26], which can also lead to contamination easily during BAL sampling. As for bacterial diversity, Chao 1 and Shannon indexes in TB lesions were both higher than those in NT samples. However, bacterial Chao 1 index in TB sputum samples is higher than that in normal controls, while the Shannon index is inverse [11]. In BAL, the Shannon index in TB group is higher than that in healthy group, while the Chao 1 index is inverse [27]. These results provided a new insight of microbial profile and diversity in the lower respiratory tract using lung tissues, instead of sputum and BAL samples.

Clinical symptoms and bacterial communities in the lower respiratory tract of inter-individual TB patient vary largely [28–30]. We observed a strong correlation of bacterial profiles in TB lesions with the status of MTB excretion among pulmonary TB patients. Interestingly, sputum microbial composition and diversity were also reported to be associated with local lung environment indicated by abnormal chest x-ray findings [12]. Specific OTUs and indicator genera detected both suggested different micro-ecological environments of TB lesions. Co-occurrence patterns of bacteria in TB-E samples showed a “small-world” property compared to TB-NE group, indicating that bacterial community in TB-E were closely connected with each other. Bacterial composition is mainly determined by the physiological and chemical environments of TB lesions [23]. When the status of TB lesions changes from MTB excretion to non-excretion, the newly formed ecological niches in TB lesions not only selectively harbor a variety of taxonomically distinct inhaled bacteria but also change the relative reproduction rates of inherent bacterial members [7, 31]. Additionally, extremely rare taxa (< 0.1% proportion) can become dominant or reach a much higher abundance in new condition [32, 33]. These results collectively revealed a strong association of lung bacteriomes in TB lesions with local lung environment and sputum culture results.

Revealing characteristics of bacterial composition in TB lesions is important to enrich our knowledge of bacterial resources in human host. Bacterial members generally exhibit complex interactions and metabolic exchanges with each other ubiquitously [34]. Despite the bacterial diversity, bacterial functions also should not be overlooked. However, microbial functional profiles are decoupled from taxonomic composition, and specific bacterial functions are strongly controlled by the external environment [23]. Broadly distributed bacterial functions identified in TB lesions, such as energy metabolism, environmental and information processing, genetic information processing, and cellular processing, were strongly conserved in most bacterial taxa, making pulmonary micro-ecological systems more resistant to changes in taxonomic composition and diversity [35, 36]. However, multiple MetaCyc functions were also enriched in TB-E and TB-NE samples, respectively. We mainly emphasized those bacterial functions with statistically different enrichments between TB-E and TB-NE samples.

In TB-E samples, bacterial MetaCyc functions responsible for menaquinone biosynthesis and chorismate biosynthesis that were significantly enriched (Supplemental Fig. 1) have been reported to impact the survival of MTB bacillus [37–40]. Menaquinone is a small molecule and possesses the capacity to signal redox status, which makes it an essential factor in triggering the persistence of MTB [37]. Menaquinone biosynthesis is important to generate energy to sustain the growth of persistent state-like MTB [38]. In TB-E lesions, enrichment of menaquinone biosynthesis in bacteria can promote MTB into the persistent state, which can lead to negative sputum culture results. The second is MetaCyc pathway responsible for chorismate biosynthesis. After the formation of chorismate, a branching of shikimate biosynthetic pathway occurs. Shikimate pathway enzymes are essential for the biosynthesis of amino acids, phenylalanine and tyrosine in bacteria, fungi, algae, and plants [39]. The enrichment of chorismate biosynthesis reflects a stronger metabolic activity of organisms, including MTB bacillus, to scavenge for nutrients from the human host in TB lesions. Chorismate has also been considered as an attractive target for developing antibiotic agents for MTB bacillus [40]. It also should be noted that the two bacterial functions can be predicted both from mycobacteria and co-existing genera based on the MetaCyc database. These discoveries provided new insights for us to elucidate the potential functions of lung bacteriomes on growth and persistent state of MTB bacillus.

Interestingly, the MetaCyc pathway responsible for penicillin resistance was also enriched in TB-E samples (Supplemental Fig. 1), although penicillin was ineffective in anti-TB treatment. According to the MetaCyc database (metacyc.org), phyla *Actinobacteria* and *Firmicutes* are expected to possess the pathway responsible for penicillin resistance. Relative abundance of *Actinobacteria* and *Firmicutes* in TB-E were 22.0% and 9.2%, which were higher than those (*Actinobacteria* 17.8% and *Firmicutes* 8.4%) in TB-NE samples. This might be the main reason that the bacterial pathway responsible for penicillin resistance was relevant to TB-E samples. The mechanism causing penicillin resistance in bacteria is overexpression of low-affinity penicillin-binding proteins (PBPs) and β-lactamases [41]. The enrichment of this bacterial pathway may indicate that TB-E lesions are more likely to co-infect with other penicillin-resistant pathogens.

In TB-NE samples, bacterial MetaCyc pathway responsible for heme b synthesis was significantly enriched. Heme biosynthesis is generally present in the host, but the bacterial metabolic pathway HEME-BIOSYNTHESIS-II is also possessed in some bacterial members under aerobic condition according to the MetaCyc database. Heme is a vital iron-containing prosthetic group for hemoproteins in MTB bacillus to acquire iron nutrient from the host. Heme b biosynthesis is one of the metabolic pathways responsible for iron assimilation [42]. A previous study has reported that macrophages can ingest bacterial components [8], which might elevate the intracellular iron level of macrophages through absorbing bacterial irons. Elevated level of intracellular iron is a character of macrophage necrosis induced by MTB infection [43, 44]. Collectively, we thought lung bacteria, especially their ingested components in macrophages, might contribute to TB pathology through ferroptosis.

As for the remaining bacterial MetaCyc functions with statistically different enrichments between TB-E and TB-NE groups, their potential impacts on MTB bacillus or pulmonary TB disease have not been clearly reported yet, but they still provide useful foundation for future investigations.

## Conclusion

This study innovatively demonstrates the characteristics of bacteriomes in lower respiratory tract using lung tissues. Bacterial composition and MetaCyc functions in TB lesions are strongly associated with the status of MTB excretion. Co-existing bacteria found in TB lesions can partially impact the survival of MTB and the development of TB pathology through metabolic functions. This study provide important bacterial information to develop novel strategies for prevention and treatment of pulmonary TB disease in the future.

## Supplementary Information

**Additional file 1:** **Supplemental Table 1.** Values of bacterial MetaCyc pathways of all TB lesions (40 TB-E and 42 TB-NE).

**Additional file 2:** **Supplemental Table 2.** Bacterial metabolic pathways that are significantly down/up regulated in TB-E compared to TB-NE.

**Additional file 3:** **Supplemental Figure 1.** Boxplots showing the abundance of Menaquinone biosynthesis, Chorismate biosynthesis, Penicillin resistance and Heme biosynthesis in TB-E and TB-NE samples.

## Data Availability

The 16S rDNA raw data were deposited in the National Center for Biotechnology Information (NCBI) at Sequence Read Archive (SRA) under BioProject accession number PRJNA747828.

## Abbreviations

TB: Tuberculosis
MTB: Mycobacterium tuberculosis
TB-E: TB patients excreting bacteria
TB-NE: TB patients not excreting bacteria
NT: Normal lung tissues
PICRUSt: Phylogenetic investigation of communities by reconstruction of unobserved states
ANOSIM: Analysis of similarity
WHO: World Health Organization
BMI: Body mass index
MAIT cells: Mucosal-associated invariant T cells
BAF: Bronchoalveolar lavage fluid
FFPE: Formalin fixed and paraffin embedded
AFB: Acid Fast Bacilli
HE: Hematoxylin–eosin
OTU: Operational taxonomic unit
PCoA: Principal coordinates analysis
ANOVO: Analysis of variance
PBP: Penicillin-binding proteins
