# Supplementary material for: Bacteriomes in lesions of pulmonary tuberculosis and its association with status of Mycobacterium tuberculosis excretion
Source: BMC Microbiol. 2022 Nov 23;22:280. doi: 10.1186/s12866-022-02698-5 (PMC9686068; doi:10.1186/s12866-022-02698-5)
Supplement: Supplementary file 3 — Additional file 3: Supplemental Figure 1. Boxplots showing the abundance of Menaquinone biosynthesis, Chorismate biosynthesis, Penicillin resistance and Heme biosynthesis in TB-E and TB-NE samples. [file 12866_2022_2698_MOESM3_ESM.docx]

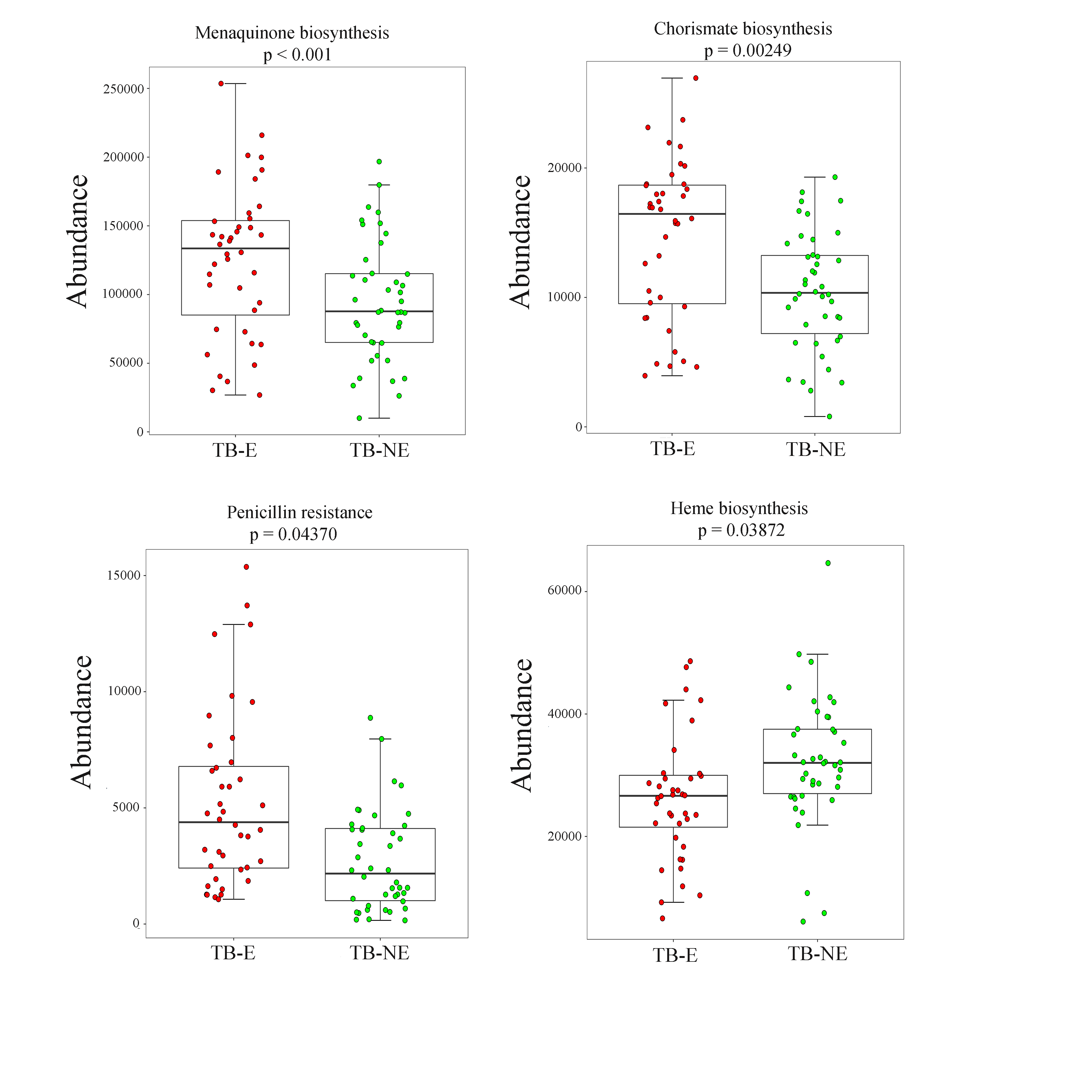


Supplemental Figure 1. Boxplots showing the abundance of Menaquinone biosynthesis, Chorismate biosynthesis, Penicillin resistance and Heme biosynthesis in TB-E and TB-NE samples.
